# Case of Pleuro-Pneumonia, Followed by Pleuritic Abscess

**Published:** 1853-12

**Authors:** S. B. Hunt


ART. III.— *Case of Pleuro-Pneumonia, followed by Pleuritic Abscess.* By S. B. Hunt, M. D.

It has for some time been my intention, to report for the pages of the Journal, the case following. It deserves notice for several reasons. The pneumonic inflammation was unusually extensive and severe; while the pleuritic symptoms were mostly concealed from observation. The character of the effusion was unexpected, and it occurred without any of those symptoms which have been usually considered indicative of it. Finally, the rapidity and the completeness of the recovery, are also unusual.

These are the main facts illustrated in its pathology. In its treatment, too, will be noticed an unusually trivial medication, while the result of the case justifies the inference that it was amply sufficient for the guidance of the cure. Another fact may be not improperly mentioned: The case requiring an expectant treatment, it was necessary that the friends of the patient should be intelligent and firm, in order to secure its continuance. It is no easy matter for parents to watch the progress of disease in an only son with folded hands. The expectant treatment rarely satisfies solicitous friends. There is something consolatory to them in the feeling that they "are doing everything," and therefore they wish to see an abundance of drugs *en route* for the patient's stomach. But in this case the parents submitted to endure all the anxiety of many months, to resist the importunities of officious friends, and to give to their physician all that confidence which was so necessary to success. It is for this reason that I mention them. It is so common to see in our Journals complaints of the interference of friends, that a word of praise bestowed upon them will have, at least, the merit of novelty.

*Dec.*, 1852. Edward Allen, aged 16 years, came home from boarding-school to spend the Christmas holidays. About the 24th I was called to him, and found him suffering with sore throat and cough. By the 26th the symptoms of pneumonia of the right lung were developed, with medium severity. He had cough, bloody expectoration, and an active grade of fever. The usual treatment was made use of. He took calomel in small doses, and was thoroughly nauseated with tartarized antimony. The acetate of ammonia was given largely, and blisters applied to the side. On the 28th there was an amelioration of the symptoms, and physical exploration indicated that the disease was to stop at its usual line of self-limitation, viz., the center of the middle lobe. His pulse had been frequent and soft, and no blood had been let from the arm. On the 29th there was an accession of inflammatory action. At this time the original seat of inflammation was solidified, and now all above it, even to the apex, was involved. The crepitant râle was very distinct in the clavicular region. The rational signs became alarming; there was some pleuritic pain, the countenance was anxious, pulse 140 per minute, expectoration very viscid, and a tendency to coma manifested. He was very restless, and groaned constantly, but made little complaint of pain. The treatment was increased in activity, so far as the strength of the patient would allow. Blistering particularly was thoroughly applied. I have omitted to mention that we had a constant and profuse perspiration, which is, I believe, a not uncommon occurrence, even in sthenic pneumonia.

On the 31st, Prof. E. M. Moore, of Rochester, saw him in consultation. Notwithstanding the very unfavorable appearances, Dr. M. gave a favorable prognosis, on the ground that as the whole right lung was now involved in hepatization, without a prospect of an immediate fatal result, we might argue that the symptoms being already as bad as they could be, would grow better instead of worse, unless the left lung should take on disease. The result justified this opinion. As to treatment: the tartar emetic had commenced running off by the bowels, (although guarded by laudanum,) in frequent watery stools. It was therefore discontinued. It is not easy to describe the treatment which followed. In accordance with Dr. Moore's advice, it was a *pro re nata* treatment, changing almost hourly with the varying indications. Stimulants were very badly borne, and evacuants were contra-indicated. On the 1st of January, 1853, delirium came on, and continued with great violence for forty-eight hours. On the 3d there was an amendment, and the case progressed toward recovery. Respiratory murmur was again heard in the apex of the lung, and day by day extended downward, until about the 10th, when the lung not clearing itself below the fourth rib anteriorly, a careful examination resulted in the diagnosis of pleuritic effusion. The relative measurement of the sides were: right side 16¼ inches, left side 15 inches. The other signs were: obliteration of intercostal depressions, entire absence of respiratory murmur, ægophony at about the fifth rib, respiration of opposite side noisy and peurile, vocal fremitus absent in lower part of the side, and entire flatness on percussion. Against the ordinary rule, however, the patient preferred to lie upon the sound side. The cough was pleuritic.

The treatment attempted was a combination of digitalis, squill, and calomel.

℞ Digitalis, grs. xii.
Squill, grs. xxxiv.
Calomel, grs. vi.

One three times daily. M. Divide into 12 powders.

After taking it a few days, a most alarming prostration occurred. The pulse went suddenly down from 120 to 90, and there was some delirium. After this, I relied on a simple supporting treatment. His nervous susceptibility was too great for blistering, and I used a liniment of tinct. digitalis, tinct. scillæ, and ol. olivar., $\overline{aa}$ equal parts, over the chest.

On the 7th of February, Dr. Moore saw him again in consultation. Dr. Moore confirmed my diagnosis. One symptom occasioned some discussion. The pulse, after recovering from the digitalis, went back to its old number, 120 per minute, and there continued. Did this mean pus instead of serous effusion? If so, why had we never had chill, or hectic, or night sweats, or diarrhœa? All these symptoms were, and had been absent, and we both concurred in considering it as a case of simple serous effusion. The patient's appetite was returning, the distention of the side decreasing. On the 21st of February, the sides measured 15 × 15$\frac{3}{4}$ in. On the 5th of March, the measurement was, right side 15$\frac{1}{2}$, and left side 15 inches. This was probably the normal size of the chest. Notwithstanding this diminution in size, there was no corresponding improvement in symptoms. Finally, about the middle of March, a little tumor pointed low down in the front of the chest, which I punctured, and it was then demonstrated that the effusion was purulent, and a very large collection of healthly looking pus was discharged. The discharge continued very profusely. He took a little quinine, but he disliking it, it was discontinued. For a while he took Huxham's tincture of bark, for the purpose of promoting his appetite, which had flagged under the discharge. Finally, all treatment was abandoned, and the discharge left to run out freely without hindrance, and a generous diet with ale prescribed.

Early in May, Dr. Moore saw him for the third time. Just then our patient was emaciated, his appetite feeble, feet swollen, and some indications of hectic. The discharge from his side was profuse, and his cough, though pleuritic in character, was, on some days, very constant and exhausting. It was decided to continue to nourish, and support. It is proper to mention here, that throughout this case, all the suggestions of myself or Dr. Moore, were carried out with zeal and judgment. The best ales were provided, the diet was carefully kept up, (*fat* food particularly being directed,) his exercise, (consisting of a daily ride in a carriage for two or three miles,) was never omitted, and the bathing, ventilation, heat of room, *cheerfulness*, and other hygienic measures were unfailingly attended to.

From this time he gradually amended. No attempt was ever made to close the external opening, and the pus was allowed to drain away, *ad libitum.* The opening closed spontaneously once or twice, but soon opened again. Early in June it closed finally, and he began to walk about the streets. He had the usual lateral curvature of the spine, and his right side was now much fallen in. In the confusion of my removal from Mendon, I have lost a portion of my notes of this case, which will explain the indefiniteness of the latter dates. This is unimportant, but I have also lost my last measurement, and only recollect that the left side then measured 1$\frac{1}{4}$ inches *more* than the right. It will be recollected that at the period of greatest effusion this measurement was reversed, the right side measuring 1$\frac{1}{4}$ inches most, thus making an extreme of difference of 2$\frac{1}{2}$ inches.

After an absence of six weeks, I had the pleasure of again seeing my patient on the 23d of July, ult. His sunburned countenance, and firm, heavy voice, indicated a complete return to health, which inquiry confirmed. In his own words he was "perfectly well." Age had undoubtedly a great influence for good in this case. But to the hygienic portion of the treatment is to be attributed the rapidity of the cure. The remarkable features of this case are:

1st. The concealed form of the pleuritis, at first.

2d. The occurrence of purulent deposit, without the usual premonitory symptoms of chill, etc.

3d. The extent of the abscess, the quantity of the discharge, (which was very great,) and the rapidity of his restoration to health.

4th. The fact that from the time when the prescription containing digitalis produced such unpleasant effects, the treatment was almost entirely hygienic, while the result proved, that the powers of nature, associated with youth, were sufficient for the cure of so great a disease involving so important an organ.

All such lessons are valuable. In the disease in question, the recoveries are the exception, the deaths are the rule. In it there is a strong temptation to over-medication. So far as a single case can prove anything, this case proves that the proper light in which to view pleuritic abscess, is as an exhausting discharge, which must work its own cure, and that the treatment should be directed to the sustentation of the vigor of the digestive organs, and the supply of the fat globule, of which so much is wasted in a long continued purulent discharge.

Since writing the above, I have had (Oct. 25th) another opportunity of seeing my patient. The curvature of the spine was much less, and the general health very good. The left side measures now 15 inches; the right 15¾ inches. As he had gained flesh, I had expected to find the measurement larger. It is probable that the left side was somewhat dilated from the time of solidification of the right lung, and that the uniformity of the measurement of that side is owing to a decrease of the dilatation, as the right lung assumed its function, corresponding to the amount of flesh gained during the process.

